# Sleep Disruption and Depression, Stress and Anxiety Levels in Women With Polycystic Ovary Syndrome (PCOS) During the Lockdown Measures for COVID-19 in the UK

**DOI:** 10.3389/fgwh.2021.649104

**Published:** 2021-06-04

**Authors:** Chris Kite, Lou Atkinson, Gordon McGregor, Cain C. T. Clark, James E. Brown, Ioannis Kyrou, Harpal S. Randeva

**Affiliations:** ^1^Centre for Active Living, University of Chester, University Centre Shrewsbury, Shrewsbury, United Kingdom; ^2^School of Biosciences, College of Health and Life Sciences, Aston University, Birmingham, United Kingdom; ^3^Warwickshire Institute for the Study of Diabetes, Endocrinology and Metabolism (WISDEM), University Hospitals Coventry & Warwickshire National Health Service (NHS) Trust, Coventry, United Kingdom; ^4^School of Psychology, College of Health and Life Sciences, Aston University, Birmingham, United Kingdom; ^5^Department of Cardiopulmonary Rehabilitation, Centre for Exercise & Health, University Hospitals Coventry & Warwickshire National Health Service (NHS) Trust, Coventry, United Kingdom; ^6^Centre for Sport Exercise & Life Sciences, Coventry University, Coventry, United Kingdom; ^7^Warwick Clinical Trials Unit, Warwick Medical School, University of Warwick, Coventry, United Kingdom; ^8^Centre for Intelligent Healthcare, Coventry University, Coventry, United Kingdom; ^9^Aston Medical Research Institute, Aston Medical School, College of Health and Life Sciences, Aston University, Birmingham, United Kingdom; ^10^Division of Translational and Experimental Medicine, Warwick Medical School, University of Warwick, Coventry, United Kingdom

**Keywords:** polycystic ovary syndrome, COVID-19, lockdown, sleep, anxiety, depression, stress, quality of life

## Abstract

**Background:** Lockdown measures have been enforced globally in response to the COVID-19 pandemic. Given the comorbidity burden in women with polycystic ovary syndrome (PCOS), these lockdown measures may have a particularly negative impact on sleep health, quality of life (QoL), and depression/stress levels in this population. The aim of this study was to explore whether such potential problems were present in women with PCOS during the COVID-19 lockdown in the UK.

**Methods:** UK women with PCOS were recruited through social media into a cross-sectional study during the COVID-19 lockdown. The study survey was delivered online, and included demographic and COVID-19 relevant questions, as well as validated questionnaires/scales, namely the Insomnia Severity Index (ISI), Depression Anxiety and Stress Scale (DASS-21), and PCOSQOL questionnaire.

**Results:** Three hundred and thirty-three women with PCOS [median age: 30.0 (9.0) years] were recruited. Participants were dichotomized based on responses regarding the impact of COVID-19 restrictions on their sleep [negative (*N* = 242) vs. no/positive (*N* = 91) impact]. No differences were noted between groups regarding age, time since PCOS diagnosis, body mass index, or number of comorbidities. Based on the ISI, 44.2% of participants reporting a negative impact on sleep exhibited at least moderately severe clinical insomnia. Compared to those who reported no/positive effect on sleep, the participants reporting a negative impact on sleep also reported poorer QoL, based on the total PCOSQOL score, with a greater impact of PCOS and poorer mood in the corresponding PCOSQOL domains. Based on the DASS-21, the latter also had statistically higher depression and stress levels compared to the former. Finally, for this cohort significant inverse correlations were noted between the ISI and PCOSQOL scores (total and domain scores), whilst the DASS-21 and ISI scores were positively correlated (all *p-*values <0.001).

**Conclusion:** The majority of recruited UK women with PCOS reported that the COVID-19 lockdown had a negative impact on their sleep, which was also associated with impaired QoL and higher depression/stress levels. Whilst further research is required, women with PCOS should be considered a vulnerable population that may experience an adverse impact on sleep, QoL and mental health well-being due to lockdown measures during the COVID-19 pandemic.

## Introduction

Coronavirus disease 2019 (COVID-19), caused by the severe acute respiratory syndrome coronavirus-2 (SARS-CoV-2), usually manifests as a respiratory tract infection with mild symptomatology (asymptomatic in many cases) (1–3). However, COVID-19 can also lead to severe manifestations in a proportion of high-risk individuals with respiratory and/or extra-pulmonary symptoms/complications requiring hospitalization (1–3). As the latter may require intensive care unit (ICU) support and may even be fatal, the COVID-19 pandemic has resulted in enforcement of varying degrees of nationwide lockdown, quarantine and self-isolation measures in many countries worldwide (4, 5). These measures aim to reduce SARS-CoV-2 transmission in the general population, and thus the risk of severe COVID-19 in vulnerable groups (e.g., older individuals and patients with certain respiratory and cardio-metabolic diseases) (6–12). Indeed, compelling evidence strongly indicates that certain chronic cardio-metabolic diseases, including diabetes and obesity, constitute key risk factors predisposing to severe COVID-19 (6–12). Notably, although severe COVID-19 is more common in men (13, 14), the aforementioned cardio-metabolic comorbidities, which significantly increase the risk of adverse COVID-19 related clinical outcomes, are also markedly prevalent in women with polycystic ovary syndrome (PCOS) (15–17).

PCOS is the most common endocrine disorder in reproductive-aged women, affecting up to 15–20% of this female population depending on the studied population and the applied diagnostic definition (18, 19). After excluding other endocrinopathies with similar symptomatology (19, 20), PCOS is typically diagnosed based on the presence of at least two out of three diagnostic criteria, namely ovulatory dysfunction, hyperandrogenism (clinical and/or biochemical) and polycystic ovaries (PCO) as identified by ultrasound (21). In addition, women with PCOS are also at a high risk of cardio-metabolic complications, particularly obesity, insulin resistance, type 2 diabetes (T2DM), hypertension, non-alcoholic fatty liver disease (NAFLD), and obstructive sleep apnoea (OSA) (22–27). Furthermore, women with PCOS often exhibit psychological comorbidity, with higher prevalence of coexisting anxiety and/or depression (28–31), which also tends to impair their overall quality of life (QoL) compared to women without PCOS (32, 33). Given this increased comorbidity burden, women with PCOS may experience a particularly negative impact from the lockdown/quarantine and self-isolation measures imposed against the transmission of SARS-CoV-2 and may also be at increased risk of severe COVID-19 (16).

Overall, global measures to control the COVID-19 pandemic are expected to inevitably have a negative psychological effect upon the general population (34) due to various factors, including the enforced quarantine measures (35) and their socio-economic impact (36), as well as the concern about COVID-19 (34). This has been previously reported in relation to SARS, which was shown to promote increased stress, anxiety and depression in the general population (37). Such findings are increasingly reported during the current COVID-19 pandemic, with an observed increase in self-reported symptoms of anxiety, depression, and stress (38). Moreover, these negative psychological effects are likely linked to disrupted sleep quality (39). In previous longitudinal studies, new onset mental health issues have been linked to increased sleep disruption (40–42), whilst disturbed sleep is also considered to be a contributing factor to the development of new mental health disorders (43). Of note, irrespective of the COVID-19 pandemic, sleep disturbances are more prevalent in women with PCOS than in the general population (44, 45), potentially due to coexisting OSA, particularly in women with poorer metabolic profiles (26), and/or depression which is also a major predictor of poor sleep quality (46). Furthermore, reduced self-esteem and body satisfaction, which are both frequently associated with PCOS, have also been demonstrated to contribute toward disrupted sleep (44).

In this context, it is likely that a 2-fold effect of increased psychological distress due to the COVID-19 pandemic alongside the established disease-related burden of PCOS may further contribute to impaired sleep quality and QoL, linked also to increased anxiety, depression, and stress levels (47). To date, there have been no published studies exploring these issues in women with PCOS during the COVID-19 pandemic. Therefore, the aim of the current study was to determine whether the COVID-19 pandemic has had a negative effect upon the sleep quality of women with PCOS in the United Kingdom (UK), and whether any such impairment was associated with reduced QoL and increased stress, anxiety, or depression in this female population.

## Methods

For the purposes of this study, we conducted a cross-sectional study based on a web-based survey between 2nd June and 17th August 2020. Ethical approval was granted by the ethics committee of Coventry University (application number: P106195) in May 2020. Recruitment was conducted through social media and with the support of Verity (the UK PCOS charity) and PCOS support groups on Facebook. Participant eligibility criteria included female sex with a previous medical diagnosis of PCOS, age from 18 to 45 years, and UK residency.

A range of structured and validated questionnaires were used to collect the study data; questionnaires were completed online using the survey software, Qualtrics XM (Qualtrics XM, Provo, Utah, USA). Where participants expressed an interest in participation, a study URL link to the survey was emailed to them directly. Alternatively, participants could access the questionnaires directly *via* the same study URL posted on social media channels. The study URL link contained an initial participant information sheet, as well as provision of informed consent. Where participants failed to complete the provided online survey, this was translated as withdrawal from the study and all data for these participants were excluded from the final analyses.

To capture relevant participant characteristics, a demographics questionnaire was created for this survey. This included self-reported information about time since PCOS diagnosis and diagnosed PCOS phenotype, height, weight, age, questions relating to sleep affected by COVID-19 restrictions (i.e., “*to what extent do you believe that quarantine measures due to COVID-19 have affected your sleep pattern?*”), presence of comorbidities and typical sociodemographic questions. In the context of this study, the PCOSQOL was utilized as a validated measure of QoL (48). The PCOSQOL is a disease-specific questionnaire which was developed and validated to measure QoL in UK women with PCOS, and is also the first PCOS-specific measurement tool to encompass all phenotypic subgroups according to the most recent diagnostic criteria (48). Briefly, this questionnaire incorporates 35 Likert-based questions allowing participants to report the impact of various PCOS-related symptoms upon their day-to-day life, with subscales which allow reporting of the impact of PCOS, infertility, hirsutism, and mood upon QoL (49–51). Total scores are summated with lower scores indicative of poorer QoL.

Information about the mental health well-being of each participant was also captured using the Depression, Anxiety and Stress Scale (DASS-21) (52). The DASS-21 includes 21 questions, seven relating to each domain for depression, anxiety and stress, and requires respondents to rate their level of agreement (0–3) to a series of statements. Each domain score is calculated by summing the responses and multiplying by two, whilst normative and cut-points for depression, anxiety, and stress are provided. Finally, the Insomnia Severity Index (ISI) was applied to measure participants' self-perceived insomnia (53), as a validated and reliable tool for the assessment of insomnia severity (54, 55). The ISI targets the subjective symptoms and consequences of insomnia, as well as the degree of concerns or distress caused by those difficulties. The ISI is composed of seven items that, respectively, evaluate the severity of sleep-onset (initial), sleep maintenance (middle), early morning awakening problems (terminal), satisfaction with current sleep pattern, interference with daily functioning, noticeability of impairment attributed to the sleep problem, and level of distress caused by the sleep problem. Each of these items is rated on a five-point Likert scale and the time interval is “*in the last 2 weeks*.” Total ISI scores range from 0 to 28, with higher scores indicating greater insomnia severity.

### Statistical Analysis

Statistical analysis was completed in IBM SPSS Statistics for Windows (Version 26.0, IBM Corp; Armonk, NY) and in R statistical software [(56), using the *car:* and *MASS:* packages] (57, 58), and statistical significance was set at *p* < 0.05. Descriptive characteristic reports were generated and Shapiro-Wilk tests of normality were completed. Accordingly, a non-parametric approach was adopted for subsequent analysis, as appropriate. Responses to the question “*to what extent do you believe that quarantine measures due to COVID-19 have affected your sleep pattern?*” were split into a dichotomous response—namely, into negative effects or no effect/positive effects—which was used as a categorical variable. Independent samples Mann-Whitney *U*-tests were completed to evaluate the between group differences. Spearman's correlations between the ISI and other questionnaires (i.e., PCOSQOL total and domain scores, and the DASS-21) were also completed for the entire study cohort and also within each group.

Given the Likert scale nature of the data in this study, where Likert scales are a special case of ordinal data, we utilized an ordinal logistic regression (OLR) approach. The OLR approach is comparable to a conventional multiple regression approach, where there may be one dependent variable and one or more independent variables. It does however differ from ordinary least squares multiple regression, by treating the dependent variable as an ordered categorical variable, based upon the principle of cumulative-odds (59). The coefficient of determination, *R*^2^, summarizes the proportion of variance in the dependent variable associated with the independent variables, with larger *R*^2^ values indicating that more of the variation is explained by the model, to a maximum of 1. However, in non-parametric regression, it is not possible to compute a traditional *R*^2^, and a pseudo *R*^2^ is computed instead. In this study, we opted to report Nagelkerke's *R*^2^ (R^2^_N_), which is an adjusted version of the Cox & Snell *R*^2^ that adjusts the scale of the statistic to cover the full range from 0 to 1 (60, 61). To test the statistical significance of each model coefficient (β), we used the Wald test to compute a Wald statistic with a chi-square distribution. All participant background characteristics were adjusted for in the OLR.

## Results

In total, 333 participants met the eligibility criteria and consented to participate in the present study, completing the online survey. Pertinent demographics/characteristics for the recruited study cohort are presented in Table 1. The vast majority of the study participants (92.5%) were of White ethnic background. Of the recruited cohort, 40% were married and a further 40% were single, with the remaining 20% being in other non-married relationships, divorced, separated or widowed. Approximately 73% of participants stated that they have no children. Moreover, the majority (63.1%) of the study participants were in full-time employment, 40.2% were educated to at least degree level, and 51.1% had the lowest household income (≤ £39,999). Finally, participants were asked to self-report their diagnosed PCOS phenotype; 46.5% indicated that they had all three PCOS diagnostic characteristics (i.e., hyperandrogenism, menstrual disruption, and PCO), 13.5% PCO and menstrual disruption, 9.6% PCO and hyperandrogenism, 6.9% hyperandrogenism and menstrual disruption, and the remaining 23.1% were unsure of the PCOS phenotype with which they had been diagnosed.

**Table 1:** Breakdown of socioeconomic and ethnicity characteristics of interest for the study cohort of UK women with polycystic ovary syndrome [PCOS; *N* = 333; median age (interquartile range) = 30.0 (9.0) years].

**Variable**	***N* (%)**
**Ethnicity**	
White	308 (92.5)
Mixed background	9 (2.7)
Asian or Asian British	8 (2.4)
Black or Black British	4 (1.2)
Other ethnic background	3 (0.9)
Declined to indicate	1 (0.3)
**Relationship status**	
Single	134 (40.2)
Married	132 (39.6)
Co-habiting	22 (6.6)
Long-term relationship	19 (5.7)
Civil-partnership	12 (3.6)
Engaged	8 (2.4)
Divorced	3 (0.9)
Separated	2 (0.6)
Widowed	1 (0.3)
**Children**	
No	242 (72.7)
Yes	91 (27.3)
**Education**	
Undergraduate	135 (40.2)
College	107 (32.1)
Postgraduate	60 (18.0)
Secondary	26 (7.8)
Doctorate	5 (1.5)
**Employment**	
Full-time employment	210 (63.1)
Part-time employment	47 (14.1)
Student	27 (8.1)
House person	19 (5.7)
Unemployed	21 (6.3)
Self-employed	9 (2.7)
**Household income**	
≤ £39,999	170 (51.1)
£40,000–£79,999	137 (41.1)
≥ £80,000	26 (7.8)

*All percentage data has been rounded to one decimal place*.

When participants' responses to the question about the impact of COVID-19 restrictions on their sleep were dichotomized into negative and no/positive responses, 242 participants reported that they had experienced either a significant or small negative effect upon their sleep, whilst the remaining 91 reported either no such effect, or at least a small positive effect upon their sleep. Using the ISI scoring guidelines, 44.2% of those reporting negative effects met the scoring threshold (>14) for a diagnosis of at least moderately severe clinical insomnia. Key outcomes of interest were compared between these two study groups and these findings are summarized in Table 2.

**Table 2:** Key outcomes of interest for the study cohort of UK women with polycystic ovary syndrome (PCOS) when split into a dichotomous response regarding the impact of the lockdown measures due to COVID-19 on sleep (reported negative effects vs. no or positive effect).

**Study variables/** **outcomes**	**Full study cohort of women with PCOS** **(*N* = 333)**	**Negative impact on sleep** **(*N* = 242)**	**No/positive impact on sleep** **(*N* = 91)**	***P***
Age (years)	30.0 (9.0)	29.0 (9.0)	30.0 (10.3)	0.426
Years Since PCOS Diagnosis	8.0 (9.9)	7.3 (9.8)	8.2 (12.0)	0.336
Weight (kg)	93.9 (37.9)	93.9 (37.2)	91.2 (37.5)	0.290
BMI (kg/m^2^)	34.8 (13.6)	35.0 (13.1)	34.0 (14.7)	0.322
PCOSQOL				
Total Score	101.0 (60.5)	97.0 (59.0)	114.0 (65.8)	**0.003**
Impact of PCOS	40.0 (28.5)	38.0 (26.0)	50.5 (30.5)	**0.001**
Infertility	24.0 (27.0)	23.0 (27.0)	30.5 (26.5)	0.077
Hirsutism	16.0 (18.0)	16.0 (17.0)	17.5 (20.3)	0.348
Mood	17.0 (10.0)	16.0 (9.0)	21.0 (11.0)	**0.001**
DASS-21				
Depression	18.0 (17.0)	18.0 (16.0)	13.0 (16.0)	**0.014**
Anxiety	10.0 (12.0)	12.0 (12.0)	10.0 (11.0)	0.094
Stress	18.0 (14.0)	18.0 (12.0)	15.0 (14.5)	**0.007**
Insomnia Severity	12.0 (9.5)	14.0 (8.0)	7.0 (8.0)	**<0.001**
Comorbidities	1.0 (2.0)	1.0 (2.0)	1.0 (3.0)	0.737

*Data are presented as median (interquartile range). Between group comparisons performed by independent samples Mann-Whitney U-tests. Insomnia severity as assessed by the validated Insomnia Severity Index (ISI). Significance was set at p < 0.05.*

*BMI, body mass index; PCOSQOL, Polycystic ovary syndrome quality of life questionnaire; DASS-21, Depression, Anxiety, and Stress Scale; P, asymptotic two-sided significance. Values in a bold font correspond to P < 0.05*.

Overall, there were no differences between the study groups regarding age, weight, body mass index (BMI), time since PCOS diagnosis, or number of comorbidities. As expected, the self-reported insomnia severity assessed by the ISI was significantly higher in those who reported a negative effect of COVID-19 on their sleep quality compared to those who reported no such effect or a relevant positive effect (Table 2). Furthermore, the former also reported poorer QoL, as measured by the total PCOSQOL score, whilst they also reported a greater impact of PCOS and poorer mood in the corresponding PCOSQOL domains. Finally, based on the corresponding DASS-21 scale scores, those reporting a negative effect of COVID-19 on their sleep quality also had statistically higher depression and stress levels, but not anxiety, compared to those who reported no such effect (Table 2).

For the entire study cohort, Spearman's correlation tests showed an inverse correlation between the ISI and PCOSQOL total score (*r*_s_ = −0.384, *p* < 0.001), and domain scores for Impact of PCOS (*r*_s_ = −0.379, *p* < 0.001), Infertility (*r*_s_ = −0.225, *p* < 0.001), Hirsutism (*r*_s_ = −0.205, *p* < 0.001), and Mood (*r*_s_ = −0.405, *p* < 0.001). Furthermore, significant positive correlations were also noted between the ISI and the DASS-21 Depression (*r*_s_ = 0.377, *p* < 0.001), Anxiety (*r*_s_ = 0.410, *p* < 0.001), and Stress (*r*_s_ = 0.467, *p* < 0.001) scores. These correlations between the ISI and the total PCOSQOL score, all PCOSQOL domains apart from Hirsutism, and DASS-21 scores also remained statistically significant within each of the two groups (data not shown).

Results of the OLR indicated that Stress, Anxiety, and Depression, as measured by the DASS-21, alongside PCOSQOL domain scores for Mood, Hirsutism, Infertility, and Impact of PCOS were significant predictors of ISI score (all *p*-values < 0.01; Table 3). Furthermore, we found that the DASS-21 variables were the greatest predictors of ISI score, accounting for the largest proportion of variance in the dependent variable [Stress: Wald χ^2^: 87.23, OR: 1.23 (1.18, 1.29), R^2^_N_: 0.05, *p* < 0.0001; Anxiety: Wald χ^2^: 64.06, OR: 1.19 (1.14, 1.25), R^2^_N_: 0.03, *p* < 0.0001; Depression: Wald χ^2^: 55.5, OR: 1.15 (1.11, 1.20), R^2^_N_: 0.03, *p* < 0.0001]. Finally, no participant characteristic significantly influenced the direction or magnitude of the OLR (all *p-*values > 0.2).

**Table 3:** Results from the ordinal logistic regression (OLR) for the study cohort of UK women with polycystic ovary syndrome (PCOS).

		**95% CI**					**95% CI**				
**Predictor**	*****β*****	**Lower**	**Upper**	**SE**	**Z**	**OR**	**Lower**	**Upper**	**Wald χ^2^**	**R^2^_**N**_**	***p***
DASS-21 stress	0.21	0.17	0.25	0.02	9.055	1.23	1.18	1.29	87.23	0.05	**<** **0.0001**
DASS-21 anxiety	0.17	0.13	0.22	0.02	7.793	1.19	1.14	1.25	64.06	0.03	**<** **0.0001**
DASS-21 depression	0.14	0.11	0.18	0.01	7.314	1.15	1.11	1.20	55.5	0.03	**<** **0.0001**
PCOSQOL mood	−0.12	−0.1	−0.08	0.01	−7.692	0.88	0.86	0.91	62.49	0.02	**<** **0.0001**
PCOSQOL hirsutism	−0.03	−0.05	−0.01	0.008	−3.288	0.97	0.95	0.99	10.98	0.005	**0.001**
PCOSQOL infertility	−0.03	−0.04	−0.01	0.006	−3.942	0.97	0.96	0.98	15.79	0.008	**<** **0.0001**
PCOSQOL impact of PCOS	−0.03	−0.04	−0.02	0.005	−6.771	0.96	0.95	0.97	48.01	0.02	**<** **0.0001**

*CI, Confidence Interval; β, beta-coefficient; SE, Standard Error; OR, Odds ratio; R^2^_N_, Nagelkerke's pseudo-R^2^; PCOSQOL, Polycystic ovary syndrome quality of life questionnaire; DASS-21, Depression, Anxiety, and Stress Scale (21 item). Values in a bold font correspond to P < 0.05*.

## Discussion

To our knowledge, this is the first study to assess the self-reported sleep quality of women with PCOS in the UK during the lockdown/quarantine measures imposed in response to the COVID-19 pandemic, and explore potential corresponding associations with QoL and depression, anxiety, and stress levels in this female population. Of note, according to the findings of the present study ~73% of the study participants reported that their sleep quality had worsened since COVID-19 restrictions were imposed. Interestingly, when compared to data from a study in the general population during COVID-19 lockdown measures (62), the prevalence of clinical insomnia based upon the ISI among the women with PCOS of the present study was markedly greater (~35 vs. ~10%). Our findings are in accord with data reported from a web-based study in the Greek general population during the national lockdown due to COVID-19, where 37.6% of participants (particularly women) scored above the threshold for insomnia based on a relevant validated questionnaire (63). Prior to the COVID-19 pandemic, global estimates for the prevalence of insomnia ranged between 3.9 and 22% (64), thus these findings suggest that there has been an exacerbation of sleep disturbances (e.g., insomnia) during this pandemic.

The present findings showing that the majority of women with PCOS self-report negative effects upon their sleep during the COVID-19 restrictions highlight insomnia and poor sleep health as a significant problem in this female population. However, it should be noted that it is difficult to determine the magnitude of this problem/change without having relevant baseline assessments before this pandemic. Indeed, due to practical difficulties in studying women with PCOS in representative population-based samples, there is an overall paucity of data on the prevalence of sleep disturbances in this population to allow precise comparisons (65). Notably, one common known sleep disorder in women with PCOS is OSA, with a recent meta-analysis reporting that OSA prevalence in women with PCOS is 35% (95% confidence interval: 22.2–48.9%) which is further increased in the presence of overweight/obesity (45). Nevertheless, OSA does not appear to be a defining factor for the findings of the present study, since only 1.2% of participants indicated that they had received a medical diagnosis of OSA, and there were no between group differences for BMI or additional comorbidities (e.g., T2DM) that are often associated with PCOS and OSA (26). Undiagnosed OSA is common in this female population (26), and may also be present among the participants of this cohort, but whether this could be an underlying factor contributing to the present findings requires further and more targeted research.

Another mechanism contributing to sleep disturbances in this study may relate to increased depression, anxiety, and stress levels. Indeed, depression, anxiety and stress yield the largest beta coefficients and account for the largest amount of variance to ISI scores in the results of the ordinal logistic regression in the present study. Of interest, when the whole study cohort was considered, 51.5% reported that they had previously received a medical diagnosis of anxiety and/or stress. Whilst it was unclear whether these diagnoses were made prior to, or during the COVID-19 pandemic, additional insight can be gained from the self-reported DASS-21 scores. Based on this validated questionnaire, the prevalence rates of at least mild depression, anxiety, and stress in the study cohort were 80.5, 70.6, and 64.6%, respectively, which are higher than the reported corresponding medical diagnoses. Notably, these are also higher than those reported by a meta-analysis for depression (33.7%; 95% CI: 27.5–40.6), anxiety (31.9%; 95% CI: 27.5–36.7), and stress (29.6%; 95% CI: 24.3–35.4) in the general population during the COVID-19 pandemic (66). Collectively, these data suggest that during the current pandemic, women with PCOS are experiencing a greater psychological burden than the general population, with a markedly higher prevalence. This is in accord with the latest international evidence-based guidance on PCOS, which highlights that, irrespective of a global pandemic, women with PCOS are more likely to experience depression and anxiety (67).

Other reported risk factors for decreased sleep quality during the COVID-19 pandemic are changes to sleep patterns (62), worries about health (68), financial consequences (69), social interactions (70), reduced physical activity (63), and gender with women being reportedly 56% more likely than men to experience sleep disruption during this pandemic (71). As such, it is plausible that such factors further contribute to a potential multifactorial effect upon an already “at risk” population (72), which clearly exacerbates sleep disruption and may result in reduced QoL. Indeed, the results of the present study support this notion, since women with PCOS who reported negative sleep effects during the COVID-19 pandemic also exhibited reduced QoL (as measured by the PCOSQOL) compared to those without any, or with positive effects on sleep. Based on the corresponding PCOSQOL domains, this association was apparently burdened by the impact of PCOS and affected mood in the study participants. However, what cannot be determined by the present findings is the directional role of sleep disruption in the aforementioned milieu for which further studies are clearly needed.

Interestingly, longitudinal studies have previously reported associations between sleep disorders, anxiety, and depression (73, 74), which are known to independently impair QoL (75). Moreover, it has been purported that there is a bidirectional relationship between sleep quality and mental well-being (76), with sleep quality independently predicting the prevalence of anxiety and/or depression, whilst anxiety and depression are also predictors for reduced sleep quality (42, 77). Due to the nature of our analysis, the current study is unable to determine the causal direction between sleep and mental well-being. However, it is likely that the COVID-19 pandemic has created an overarching environment which further exacerbates this relationship with the net result being further impairments of mental health and sleep quality leading to reduced QoL. As prior to the COVID-19 pandemic, women with PCOS were already recognized as a patient population at an increased risk of anxiety/stress, depression, sleep disorders, and impaired QoL (18, 19); the disrupting circumstances of this pandemic mean then that there should be a renewed and heightened focus from healthcare professionals to ensure that adequate support and treatment provision is available to reduce and, where possible, prevent further comorbidity and impaired QoL in these women (16).

### Study Limitations

There are certain limitations which should be acknowledged in the present study. This study relied upon participant self-report which can lead to a degree of decreased clarity/accuracy in the provided answers, thus inevitably introducing a degree of information bias to the study findings. For example, it is known that individuals tend to over-report their height and under-report their weight (78), an effect which appears to be further exaggerated in individuals with overweight/obesity (79). This may lead to discrepancies in self-reported anthropometric data which, given the key role of metabolic health in PCOS severity/comorbidity (80) and sleep quality (26) may impact to some degree on the study findings. However, this methodology is frequently employed in studies of this nature, whilst validated instruments/questionnaires were utilized to capture key study data of interest. Another study limitation is the lack of comparative baseline data for the study cohort for a period prior to the COVID-19 pandemic. The study survey questions asked about changes in sleep quality due to COVID-19 restriction measures, but without validated baseline measurements, it is not possible to quantify the exact extent to which the corresponding outcomes have been affected. This issue could be addressed to some degree at a follow-up time point when current restrictions have been eased/lifted. To this aim, the relevant prospective follow-up of this study cohort has been planned. Furthermore, although the large sample size in the present study is a distinct strength, it is accompanied by some inherent limitations. For instance, OLR yielded significant R^2^_N_ for all variables, yet most were of little practical significance; thus, in interpreting these results, the potential fallacy of large sample sizes must be considered (81). Finally, as this is a cross-sectional study, the present findings cannot be used to infer conclusions regarding the temporal/causal relationship between the noted negative impact on sleep and reported levels of depression/stress.

## Conclusion

The present study offers a novel insight regarding the self-reported sleep quality of women with PCOS during the COVID-19 pandemic lockdown, and how this is associated with QoL and depression, anxiety, and stress levels in this population. Based on our present findings, it is evident that the majority of UK women with PCOS in this study cohort feel that the applied measures imposed in response to the COVID-19 pandemic had a negative impact on the quality of their sleep, with high prevalence of insomnia. There is also evidence that those women with PCOS and impaired sleep have greater levels of psychological morbidity (e.g., depression/stress) and reduced QoL. Whilst it appears that the restrictive measures due to COVID-19 have increased this comorbidity burden in these women with PCOS, the exact magnitude of the impact of this global pandemic upon these parameters and the underlying temporal/causal relationship is less clear. Nevertheless, it has previously been reported that during such disease outbreaks, the number of individuals whose mental health is negatively affected can be greater than the number affected by the infection (82), and that the mental health implications and their prevalence can be even more significant than the epidemic itself (83). Another key message to consider based on the present study is that there are certain groups that may remain relatively overlooked despite being particularly vulnerable during this pandemic. Women with PCOS should be considered within these parameters, since they are at increased risk of cardio-metabolic complications which, in turn, may increase the risk of severe COVID-19, whilst they are also susceptible to significant psychological comorbidity which, regardless of COVID-19, may impair their overall well-being.

## Data Availability Statement

The raw data supporting the conclusions of this article will be made available, without undue reservation, by the authors upon reasonable request and where it is ethically acceptable to do so, and does not violate the protection of participants, or other valid ethical, privacy, or security concerns.

## Ethics Statement

The studies involving human participants were reviewed and approved by Coventry University. The patients/participants provided their online written informed consent to participate in this study.

## Author Contributions

CK, LA, GM, JB, IK, and HR developed the study protocol. CK drafted the initial manuscript. CK and CCTC performed the statistical analyses. CK, CCTC, LA, GM, JB, IK, and HR contributed to the literature search, drafted, and/or revised sections of this manuscript. IK and HR supervised the study, combined, edited, and revised all drafts of this manuscript. All authors approved the final manuscript.

## Conflict of Interest

The authors declare that the research was conducted in the absence of any commercial or financial relationships that could be construed as a potential conflict of interest.
